# The effect of eye protection on SARS-CoV-2 transmission: a systematic review

**DOI:** 10.1186/s13756-021-01025-3

**Published:** 2021-11-04

**Authors:** Oyungerel Byambasuren, Elaine Beller, Justin Clark, Peter Collignon, Paul Glasziou

**Affiliations:** 1 Institute for Evidence-Based Healthcare, Bond University, 14 University Dr, Robina, QLD 4226 Australia; 2 Medical School, Australian National University, Canberra, ACT Australia

**Keywords:** COVID-19, SARS-CoV-2, Eye protection, Face shield, Infection prevention

## Abstract

**Background:**

The effect of eye protection to prevent SARS-CoV-2 infection in the real-world remains uncertain. We aimed to synthesize all available research on the potential impact of eye protection on transmission of SARS-CoV-2.

**Methods:**

We searched PROSPERO, PubMed, Embase, The Cochrane Library for clinical trials and comparative observational studies in CENTRAL, and Europe PMC for pre-prints. We included studies that reported sufficient data to estimate the effect of any form of eye protection including face shields and variants, goggles, and glasses, on subsequent confirmed infection with SARS-CoV-2.

**Results:**

We screened 898 articles and included 6 reports of 5 observational studies from 4 countries (USA, India, Columbia, and United Kingdom) that tested face shields, goggles, and wraparound eyewear on 7567 healthcare workers. The three before-and-after and one retrospective cohort studies showed statistically significant and substantial reductions in SARS-CoV-2 infections favouring eye protection with odds ratios ranging from 0.04 to 0.6, corresponding to relative risk reductions of 96% to 40%. These reductions were not explained by changes in the community rates. However, the one case–control study reported odds ratio favouring no eye protection (OR 1.7, 95% CI 0.99, 3.0). The high heterogeneity between studies precluded any meaningful meta-analysis. None of the studies adjusted for potential confounders such as other protective behaviours, thus increasing the risk of bias, and decreasing the certainty of evidence to very low.

**Conclusions:**

Current studies suggest that eye protection may play a role in prevention of SARS-CoV-2 infection in healthcare workers. However, robust comparative trials are needed to clearly determine effectiveness of eye protections and wearability issues in both healthcare and general populations.

**Supplementary Information:**

The online version contains supplementary material available at 10.1186/s13756-021-01025-3.

## Background

Facial protection—for both wearer and close contacts—has been a crucial and controversial feature of the COVID-19 pandemic. For example, WHO recommends eye protection (goggles or face shield) for health care workers caring for COVID-19 patients but are not currently recommended for those caring for COVID-19 patients at home even when in the same room. A major uncertainty has been how much protection is provided by different forms and combinations of facial coverings.

We know that respiratory viruses such as SARS-CoV-2 can enter the respiratory tract via the nose, mouth, or eyes and inoculation may occur via air-to-face or hands-to-face. Early in the COVID-19 pandemic the mix of these routes was unclear, but now the air-to-face route is agreed to be an important factor in most cases. Less clear is what are the proportions of inoculation that occurs via the nose versus eyes. The cornea has ACE-2 receptors which may allow SARS-CoV-2 infection, but more likely is inoculation of the nasal epithelium via the nasolacrimal duct [1].

One of the earliest evidence for potential importance of eyes in infection transmission and therefore eye protection came from the Spanish influenza epidemic [2]. Despite evidence from the previous SARS and MERS outbreaks suggesting an impact of eye protection [3], the COVID-19 pandemic has seen much less research focused on eye protection. A call for face shields to provide eye protection early in the pandemic seemed to be largely ignored in both practice and research [4], despite some promising studies. One early observational study in India of healthcare workers dealing with COVID-19 patients in the community showed a dramatic decline in the numbers of workers getting infected after face shields were made mandatory [5]. However, this study has received relatively little attention. A recent plea in the Lancet Microbe pointed to eye protection as a potential missing key [1].

Therefore, to examine the potential contribution of eye protection, we aimed to identify, appraise, and synthesise all studies that estimated the impact of any form of eye protection including face shields and variants, goggles, glasses, and others on transmission of SARS-CoV-2.

## Method

We conducted a systematic review using enhanced processes and automation tools [6]. We searched the PROSPERO database to rule out existence of a similar review; searched PubMed, Embase, and The Cochrane Library’s CENTRAL for clinical studies, and Europe PMC for pre-prints from 1 Jan 2020 to 1 Jun 2021. A search string composed of MeSH terms and words was developed in PubMed and was translated to be run in other databases using the Polyglot Search Translator [7]. The search strategies for all databases are presented in Additional file 1: Supplement 1. We also searched World Health Organization—International Clinical Trials Registry Platform (ICTRP) and ClinicalTrials.gov databases from inception until 1 Jun 2021. Protocol for this review was developed but not registered. We used PRISMA 2020 statement as a reporting guideline for this review [8].

All publication types and languages were included in the search. We also conducted forward and backward citation searches for included studies in the Scopus citation database.

Our inclusion criteria—based on participants, interventions, and outcomes—were:

- Participants: all studies in humans, whether community or health care workers.
- Interventions: any form of eye protection, including face shields, goggles, or modified snorkel masks, with or without face masks.
- Comparators: No eye protection, with or without face masks.
- Outcomes: number of laboratory-confirmed infection with SARS-CoV-2.

We included any comparative study design (including before-and-after). We excluded studies if they did not provide sufficient data to make a comparison between eye protection and no eye protection; laboratory experiments; and any other eyewear that was not designed for prevention of respiratory virus transmission.

### Screening

**Fig. 1:**
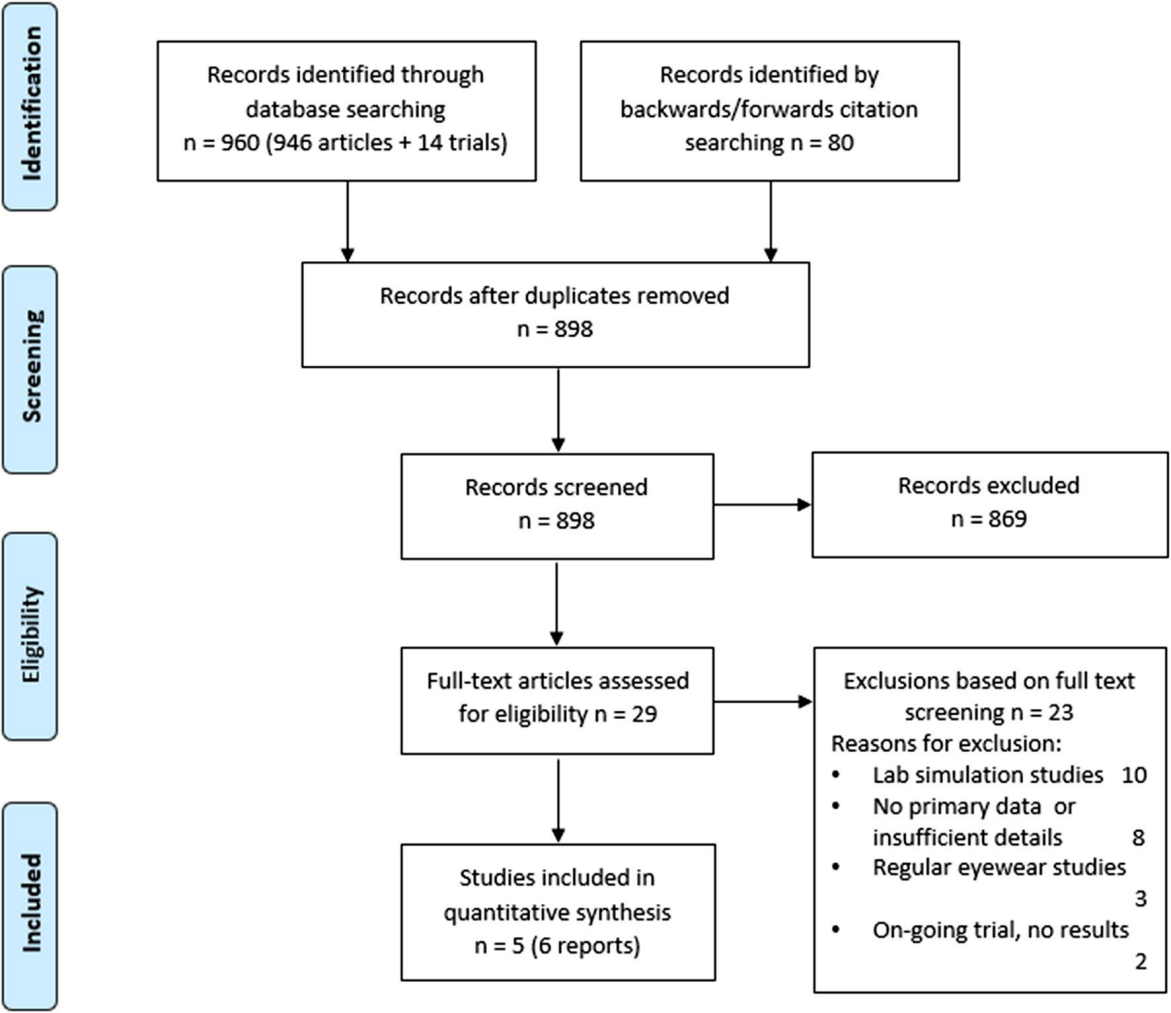
Screening and selection of articles

Two authors (OB, EB) independently screened titles, abstracts, and full texts. Full text articles were retrieved by OB. Discrepancies were resolved by referring to a third author (PG). The selection process was recorded in sufficient detail to complete a PRISMA flow diagram (see Fig. 1) and a list of excluded (full text) studies with reasons for exclusions are provided in Additional file 1: Supplement 2.

### Data extraction

Two authors independently extracted the following data from included studies:

- Study characteristics: country, study type, study setting, details of study PICO, timeframe,
- Quantitative outcomes: total number of populations tested, total number of COVID( +) cases both before and after the intervention was mandated.
- Number of cumulative COVID-19 cases in the community before and after the intervention, from external sources when necessary.

### Assessment of the risk of bias

Two authors (OB, EB) independently assessed the risk of bias for each study using the ROBINS-I for observational studies [9]. We identified the following confounding domains relevant to most studies in order of most to least likely (Table 1):

- change in risk of COVID-19 from before, during and after the intervention,
- test frequency or who is being tested (i.e., because of change in those being followed for infection),
- comparator (use or compliance with other PPE),
- setting (community, hospital, COVID-specific ward).

**Table 1:** Characteristics of included studies (n = 5)

Study ID, area, country	Study type and setting	Intervention period	Population	Intervention description	Outcomes reported
Al Mohajer [10] Texas, USA	Before-after; hospital	Jul 6–Sep 7, 2020	n = 6527 HCPs,	Face shields (Lazarus 3D, Corvallis, OR, USA). Goggles allowed as an alternative for those unable to tolerate face shields	n (%) HCPs tested positive; total patient days; hospital-acquired infections
Bhaskar [5] Chennai, India	Before-after; community screening project	May 10–Jun 30, 2020	n = 62 community health workers	Face shields made of polyethylene terephthalate (250-μm thickness)	n (%) HCPs tested positive; n (%) community dwelling people visited +
Hamilton [11] Devon, UK	Before-after; District hospital COVID ward	mid-Dec 2020–Feb 2021	n = 410 HCPs	Universal visors for all patient care	n (%) HCPs tested positive
Rodriguez-Lopez [13] Cali, Columbia	Case–control; hospital	Jun 10–Jul 25, 2020	n = 223 HCWs	Face shield or goggles	Factors associated with SARS-CoV-2 infection including use of PPE, and compliance
Shah [14] Minnesota, USA	Retrospective cohort; Clinic	May 13–Nov 30, 2020	n = 345 HCPs	Eye protection (face shield, protective wraparound eye wear, or a polycarbonate face shield or helmet)	n (%) HCPs tested positive

*HCPs* healthcare professional, *HCWs* healthcare workers

We also identified the following two co-interventions that could be different between intervention groups and that could impact outcomes: (i) Addition of other infection control measures. Particularly important in before-after studies; (ii) Frequency of testing for COVID-19 (e.g., change in policy for testing, mandating of testing).

### Data analysis

We planned to do meta-analyses when two or more studies reported the same outcome provided that the heterogeneity was sufficiently low. The forest plot of intervention effects was created using Review Manager (RevMan) Version 5.4.1 (Copenhagen: The Nordic Cochrane Centre, The Cochrane Collaboration, 2014). For dichotomous outcomes, we used the odds ratios. We did not assess publication bias / small studies effect because fewer than 10 studies were included. Where data were missing, study authors were contacted.

## Results

We screened titles and abstracts of 898 articles including 14 registered trials and assessed 29 full text articles for inclusion (Fig. 1). Main reasons for exclusion were lack of primary data and laboratory experiments. Full list of excluded studies with reasons can be found in the Additional file 1: Supplement 2. Five published observational studies (in 6 reports) from 4 countries were included in the quantitative analysis [5, 10–14] (Table 1). Al Mohajer et al. [10] is a published full version of Hemmige et al. [12], with more complete data and therefore, was included in the analysis. All included studies were conducted on healthcare professionals [10, 11, 14] (HCPs), community health workers [5], and healthcare workers (HCWs, defined as all people working in healthcare environment regardless of direct or indirect involvement in clinical activities [13]).

Description of the eye protections ranged from wraparound eyewear and goggles to full face shield or visor in addition to approved masks and other infection control measures in the corresponding clinical setting (Table 1). Eye protections were mandated to be worn upon entry to the hospital [10], or during all patient interactions [5, 11, 13, 14]. Three of the studies instituted the eye protection intervention during the rise of community cases during the first wave of infections in the respective communities [10, 11, 14]. However, none of them adjusted for change in risk of infection (e.g., community rates).

**Fig. 2:**
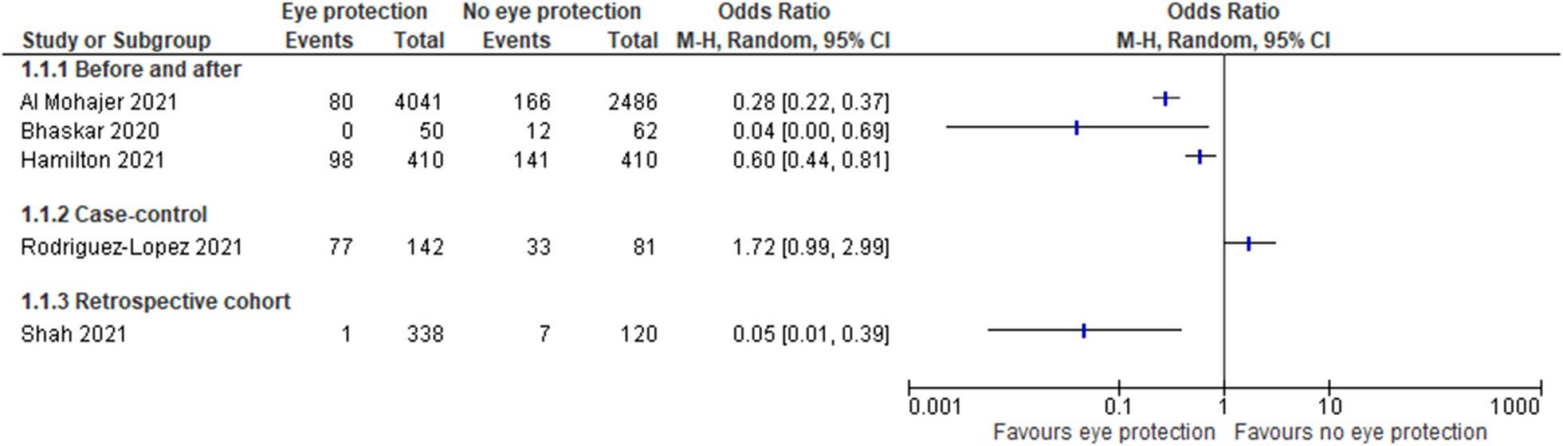
Forest plot of effect of eye protection in healthcare professionals

The high heterogeneity between studies precluded a meaningful meta-analysis. Forest plot of each study outcomes are presented in Fig. 2. Most of the studies reported reduced number of infections after instigating eye protection for patient interaction. The three before-and-after studies all showed statistically significant and substantial reductions in SARS-CoV-2 infections favouring eye protection with odds ratios ranging from 0.04 to 0.6, corresponding to risk reductions of 96% to 40% [5, 10, 11]. However, the one case–control study reported odds ratio favouring no eye protection (OR 1.7, 95% CI 0.99, 3.0) [13]. None of the studies adjusted for potential confounders such as other protective behaviours.

We also found three prevalence studies that looked at potential protective effect of regular eyewear in general population [15–17]. They suggested that wearing regular eyewear for more than 8 h a day could reduce SARS-CoV-2 infection, but there was insufficient data to establish causation.

### Risk of bias

The risk of bias of the included studies was assessed by ROBINS-I for observational studies. The risk of bias within individual studies was judged overall as moderate to serious with confounding being the primary source (see Methods). Risk of selection bias, classification of intervention, measurement of outcomes and selection of reported results were judged to be low risk of bias (Table 2).

**Table 2:** Risk of bias in included studies assessed by ROBINS-I

	Risk of bias domains							
Study ID	Bias due to confounding	Bias in selection of participants	Bias in classification of interventions	Bias due to deviations from intended interventions	Bias due to missing data	Bias in measurement of outcomes	Bias in selection of the reported result	Overall risk of bias
Al Mohajer [10]	Moderate	Low	Low	Low	NI	Low	Low	Moderate
Bhaskar [5]	NI	Low	Low	Low	Low	Low	Low	Moderate
Hamilton [11]	Moderate	Low	Low	Low	NI	Low	Low	Moderate
Rodriguez-Lopez [13]	Serious	Low	Low	NI	Low	Low	Low	Serious
Shah [14]	Serious	Low	Low	NI	Low	Low	Low	Serious

*NI* no information

## Discussion

Of the five observational studies identified, four showed substantial and statistically significant reductions in COVID-19 infections of health care workers after mandatory eye protection—mainly from face shields—was introduced; one case–control study showed an increase which was partly explained by an increase in community transmission. All five studies were non-randomized, and did not adjust for potential confounders, so the overall risk of bias was high. Therefore, the evidence summarised here is very low certainty.

One important confounder for the before-after studies is any change in community transmission between the before-after periods; as demonstrated in Fig. 2, the community rates were generally higher in the after period; hence adjustment would only increase the size of the estimated reduction. However, the higher rates may also mean increases in other protective behaviours which are not reported in any of the studies. Finally, while the studies’ main intervention was face shields, they also allowed the use of some other forms of eye protection such as goggles.

A previous review of observational studies on the effects in of eye protection in the SARS and MERS epidemics found a reduction in transmission of 66% and 76% respectively [3]. These reductions are comparable with the reductions seen in the three before-after studies of this review. Several laboratory studies using mannikins have examined the potential effects of face shields but vary greatly in their design and their application to real-world settings. One study tested facing mannikins 25 cm apart with the emitter sending an aerosol spray with particles with a range of size from less than 0.3 µm to 10 µm; they found a reduction in particles received of 55% with face shields compared to 22% for a mask, and 97% for both [18]. A study with a mannikin 60 cm from the spray found greater reductions and face shields providing greater protection than masks [19]. However, these studies were of water aerosols not transmission of viral particles and are very incomplete simulations of human interaction. Neither separated eye protection specifically from face protection.

These studies provide suggestive evidence that face shields provide some protective effect, and that this may be substantial. These studies cannot determine how much of the protective effect is due to reduction of transmission from the eyes via nasolacrimal duct to nose. Furthermore, a face shield—the main protection used—may provide additional inhalation protection as seen in some of the laboratory studies. While goggles also provide eye protection, face shields will likely give substantial protection against inhalation of droplets as well as eye protection and are more comfortable to wear. Hence face shields—in addition to masks—should be considered for higher risk situations—such as contact tracing, quarantine workers, and some primary care consultations—or when there is substantial Covid spread in the community. Additional protection is likely to be particularly important to health care workers in settings where currently only face masks are being used.

While these observational studies show an interesting potential protection from face shields as add-on to face masks, they do not clarify whether such protection is from reduced inhalation or eye protection. Trials of the incremental value of face shields in addition or instead of face masks and comparative studies of face shields and eye goggles with face masks all seem warranted. Such studies should also measure comfort and adherence of different options used, as correct and sustained usage is also critical to effectiveness.

## Supplementary Information

**Additional file 1**.** Supplement 1**. Full search strategy.** Supplement 2**. Excluded studies following full text screening with reasons.

## Data Availability

All data generated or analysed during this study are included in this published article and its supplementary information files.
