# Supplementary material for: The effect of eye protection on SARS-CoV-2 transmission: a systematic review
Source: Antimicrob Resist Infect Control. 2021 Nov 4;10:156. doi: 10.1186/s13756-021-01025-3 (PMC8567128; doi:10.1186/s13756-021-01025-3)
Supplement: Supplementary file 1 — Additional file 1. Supplement 1. Full search strategy. Supplement 2. Excluded studies following full text screening with reasons. [file 13756_2021_1025_MOESM1_ESM.docx]

## **Supplementary material for the article “The effect of eye protection on SARS-CoV-2 transmission: a systematic review”**

**Supplement 1. Full search strategy:** page 1-2

**Supplement 2. Excluded studies following full text screening with reasons:** page 3-4

**Supplement 1. Full search strategy**

**PubMed**:

("COVID-19"[Mesh] OR "SARS-CoV-2"[Mesh] OR "COVID-19"[Supplementary Concept] OR "SARS-CoV-2 variants" [Supplementary Concept] OR “COVID-19”[tiab] OR COVID19[tiab] OR “COVID 19”[tiab] OR “SARS-CoV-2”[tiab] OR “2019-nCoV”[tiab] OR “Novel coronavirus”[tiab] OR “Coronavirus 2019”[tiab] OR “Coronavirus 19”[tiab] OR “COVID 2019”[tiab] OR "2019 ncov"[tiab] OR “Wuhan coronavirus”[tiab])

AND

("Eye Protective Devices"[Mesh] OR Glasses[tiab] OR Goggle[tiab] OR “Eye protection”[tiab] OR Faceshield[tiab] OR Faceshields[tiab] OR Goggles[tiab] OR “Face shield”[tiab] OR “Face shields”[tiab] OR Visors[tiab] OR “Prophylactic measures”[tiab])

AND

("transmission"[sh] OR Prevention[tiab] OR Reduces[tiab] OR Spread[tiab] OR Efficacy[tiab] OR Reduction[tiab] OR Transmission[tiab])

AND

(“randomized controlled trial”[pt] OR “controlled clinical trial”[pt] OR randomized[tiab] OR randomised[tiab] OR placebo[tiab] OR randomly[tiab] OR trial[tiab] OR groups[tiab] OR Crossover[tiab] OR "Comparative Study"[pt] OR "Evaluation Study"[pt] OR "Epidemiologic Studies"[Mesh] OR “case-control studies”[Mesh] OR “Cohort Studies”[Mesh] OR “case control”[tiab] OR Cohort[tiab] OR “Follow up”[tiab] OR Observational[tiab] OR Longitudinal[tiab] OR Prospective[tiab] OR retrospective[tiab] OR “cross sectional”[tiab] OR “Cross-Sectional Studies”[Mesh] OR Investigated[tiab] OR Evaluated[tiab] OR Impact[tiab] OR Analysis[tiab] OR Statistics[tiab] OR Data[tiab] OR "statistics and numerical data"[sh] OR "epidemiology"[sh] OR Experimental[tiab] OR Experiment[tiab] OR "Letter"[pt] OR "Comment"[pt])

**Embase**

('coronavirus disease 2019'/exp OR 'Severe acute respiratory syndrome coronavirus 2'/exp OR COVID-19:ti,ab OR COVID19:ti,ab OR "COVID 19":ti,ab OR SARS-CoV-2:ti,ab OR 2019-nCoV:ti,ab OR "Novel coronavirus":ti,ab OR "Coronavirus 2019":ti,ab OR "Coronavirus 19":ti,ab OR "COVID 2019":ti,ab OR "2019 ncov":ti,ab OR "Wuhan coronavirus":ti,ab)

AND

("eye protective device"/exp OR Glasses:ti,ab OR Goggle:ti,ab OR "Eye protection":ti,ab OR Faceshield:ti,ab OR Faceshields:ti,ab OR Goggles:ti,ab OR "Face shield":ti,ab OR "Face shields":ti,ab OR Visors:ti,ab OR "Prophylactic measures":ti,ab)

AND

(Prevention:ti,ab OR Reduces:ti,ab OR Spread:ti,ab OR Efficacy:ti,ab OR Reduction:ti,ab OR Transmission:ti,ab)

AND

(random* OR factorial OR crossover OR placebo OR blind OR blinded OR assign OR assigned OR allocate OR allocated OR 'crossover procedure'/exp OR 'double-blind procedure'/exp OR 'randomized controlled trial'/exp OR 'single-blind procedure'/exp OR 'epidemiology'/exp OR 'controlled study'/exp OR 'cohort analysis'/exp OR "case control":ti,ab OR Cohort:ti,ab OR "Follow up":ti,ab OR Observational:ti,ab OR longitudinal:ti,ab OR Prospective:ti,ab OR retrospective:ti,ab OR "cross sectional":ti,ab OR 'Cross-Sectional Studies'/exp OR Investigated:ti,ab OR Analysis:ti,ab OR Statistics:ti,ab OR Data:ti,ab)

**Cochrane CENTRAL**

([mh "COVID 19"] OR [mh "SARS CoV 2"] OR “COVID 19”:ti,ab OR COVID19:ti,ab OR "COVID 19":ti,ab OR “SARS CoV 2”:ti,ab OR 2019 nCoV:ti,ab OR "Novel coronavirus":ti,ab OR "Coronavirus 2019":ti,ab OR "Coronavirus 19":ti,ab OR "COVID 2019":ti,ab OR "2019 ncov":ti,ab OR "Wuhan coronavirus":ti,ab)

AND

([mh "Eye Protective Devices"] OR Glasses:ti,ab OR Goggle:ti,ab OR "Eye protection":ti,ab OR Faceshield:ti,ab OR Faceshields:ti,ab OR Goggles:ti,ab OR "Face shield":ti,ab OR "Face shields":ti,ab OR Visors:ti,ab OR "Prophylactic measures":ti,ab)

AND

([mh /TM] OR Prevention:ti,ab OR Reduces:ti,ab OR Spread:ti,ab OR Efficacy:ti,ab OR Reduction:ti,ab OR Transmission:ti,ab)

**Cochrane COVID-19 Study Register**

("Eye protection" OR Faceshield OR Faceshields OR "Face shield" OR "Face shields")

AND

(Prevention OR Reduces OR Spread OR Efficacy OR Reduction OR Transmission)

**Europe PMC (Preprints)**

(COVID-19 OR SARS-CoV-2 OR COVID-19 OR COVID19 OR "COVID 19" OR SARS-CoV-2 OR 2019-nCoV OR "Novel coronavirus" OR "Coronavirus 2019" OR "Coronavirus 19" OR "COVID 2019" OR "2019 ncov" OR "Wuhan coronavirus")

AND

("Eye protection" OR Faceshield OR Faceshields OR "Face shield" OR "Face shields")

AND

(Prevention OR Reduces OR Spread OR Efficacy OR Reduction OR Transmission)

AND

(SRC:PPR)

**Supplement 2. Excluded studies following full text screening with reasons**

| **No.** | **Excluded articles** | **Reason** |
| --- | --- | --- |
| 1 | Chua JX, Ong L, Tan CH. Innovative Face Shields Help Frontliners Face-off COVID-19 Pandemic. Ann Acad Med Singap. 2020;49(12):1044-7. | No primary data |
| 2 | Gupta S, Jangra RS, Gupta S, Gujrathi AV, Sharma A. Makeshift face shield for healthcare professionals during the COVID-19 pandemic. Clin Exp Dermatol. 2020;45(6):751-2. | No primary data |
| 3 | Ha JF. The COVID-19 pandemic and face shields. Br J Surg. 2020;107(10):e398. | No primary data |
| 4 | Khunti K, Adisesh A, Burton C, Chan XHS, Coles B, Durand-Moreau Q, et al. Analysis ;The efficacy of PPE for COVID-19-type respiratory illnesses in primary and community care staff. British Journal of General Practice. 2020;70(697):413-6. | No primary data |
| 5 | MacIntyre CR, Wang Q. Physical distancing, face masks, and eye protection for prevention of COVID-19. Lancet 2020; 395(10242): 1950-1. | No primary data |
| 6 | Marra AR, Edmond MB, Popescu SV, Perencevich EN. Examining the need for eye protection for coronavirus disease 2019 (COVID-19) prevention in the community. Infect Control Hosp Epidemiol 2021; 42(5): 646-7. | No primary data |
| 7 | Hawkins ES, Fertel BS, Muir MR, Meldon SW, Delgado FJ, Smalley CM. Adding eye protection to universal masking reduces COVID-19 among frontline emergency clinicians to the level of community spread. Am J Emerg Med 2020. | No primary data/insufficient detail |
| 8 | Tan Gse LKZSMMLVSCMPBFNOTABSLY. Effect of extended use N95 respirators and eye protection on personal protective equipment (PPE) utilization during SARS-CoV-2 outbreak in Singapore. Antimicrobial resistance and infection control 2020; 9(1): 86. | No primary data |
| 9 | Ha JF, Collier R, Kop AM, Morrison DA. The COVID-19 pandemic and ENT modified face shields. Br J Surg 2020; 107(11): e448-e9. | Lab study |
| 10 | Li L, Niu M, Zhu Y. Assessing the effectiveness of using various face coverings to mitigate the transport of airborne particles produced by coughing indoors. Aerosol Science and Technology 2020; 55(3): 332-9. | Lab study |
| 11 | Lindsley WG, Blachere FM, Law BF, Beezhold DH, Noti JD. Efficacy of face masks, neck gaiters and face shields for reducing the expulsion of simulated cough-generated aerosols. Aerosol Science and Technology 2021; 55(4): 449-57. | Lab study |
| 12 | Ronen A, Rotter H, Elisha S, et al. Investigation of the protection efficacy of face shields against aerosol cough droplets. J Occup Environ Hyg 2021; 18(2): 72-83. | Lab study |
| 13 | Schmitt J, Jones L, Aeby E, Gloor C, Moser B, Wang J. Protection Level and Reusability of a Modified Full-Face Snorkel Mask as Alternative Personal Protective Equipment for Healthcare Workers During the COVID-19 Pandemic. 2020. | Lab study |
| 14 | Stephenson T, Cumberland C, Kibble G, et al. Evaluation of Facial Protection Against Close-Contact Droplet Transmission. 2021. | Lab study |
| 15 | Tretiakow D, Tesch K, Skorek A. Mitigation effect of face shield to reduce SARS-CoV-2 airborne transmission risk: Preliminary simulations based on computed tomography. Environmental Research 2021; 198. | Lab study |
| 16 | Verma S, Dhanak M, Frankenfield J. Visualizing droplet dispersal for face shields and masks with exhalation valves. 2020. | Lab study |
| 17 | Viola IM, Peterson B, Pisetta G, et al. Face Coverings, Aerosol Dispersion and Mitigation of Virus Transmission Risk. 2021. | Lab study |
| 18 | Wendling JM, Fabacher T, Pébaÿ PP, Cosperec I, Rochoy M. Experimental Efficacy of the Face Shield and the Mask against Emitted and Potentially Received Particles. Int J Environ Res Public Health 2021; 18(4). | Lab study |
| 19 | Lehrer S, Rheinstein P. Eyeglasses Reduce Risk of COVID-19 Infection. In Vivo 2021; 35(3): 1581-2. | Regular eyewear study |
| 20 | Zeng W, Wang X, Li J, et al. Association of Daily Wear of Eyeglasses With Susceptibility to Coronavirus Disease 2019 Infection. JAMA Ophthalmol 2020; 138(11): 1196-9. | Regular eyewear study |
| 21 | Saxena AK. Risk of Corona virus disease 2019 (COVID-19) among spectacles wearing population of Northern India. 2021. | Regular eyewear study |
| 22 | Nct. Effectiveness and Adherence to Closed Face Shields to Prevent COVID-19 Transmission. https://clinicaltrialsgov/show/NCT04647305, 2020. https://www.cochranelibrary.com/central/doi/10.1002/central/CN-02205986/full | On-going trial with no results |
| 23 | Nct. Enhanced Hood PPE to Minimize COVID-19 Transmission to Front-line Health Care Workers. https://clinicaltrialsgov/show/NCT04373096, 2020. https://www.cochranelibrary.com/central/doi/10.1002/central/CN-02103676/full | On-going trial with no results |
